# Supplementary material for: Sensitivity analysis on the declining population in Japan: Effects of prefecture-specific fertility and interregional migration
Source: PLoS One. 2022 Sep 14;17(9):e0273817. doi: 10.1371/journal.pone.0273817 (PMC9473415; doi:10.1371/journal.pone.0273817)
Supplement: S1 File — Text A, Representation theorem for a right eigenvector of an irreducible non-negative matrix. Text B, Theorem for infinite series expansion of characteristic equation. Text C, Original definition of type-reproduction number. Text D, Extension theorem of type-reproduction number. (ZIP) [file pone.0273817.s001.zip › Supporting_Infomation1_final.pdf]

Supporting information

Text A

Representation theorem for a right eigenvector of an irreducible non-negative matrix

Theorem A. *Let* $\mathbf{A} := (a_{ij})_{1 \le i,j \le n}$ *be an irreducible non-negative matrix. Then, the right eigenvector of* $\mathbf{A}$*,*

$$\mathbf{w} := (w(i))_{1 \le i \le n}\,, \tag{S.1}$$

corresponding to the eigenvalue $r \neq 0$*, which is equivalent to the spectral radius of* $\mathbf{A}$*, can be represented as*

$$w(i) = w(\ell)\left(r^{-1}a_{i\ell} + \sum_{m=1}^{\infty}\sum_{j_1, j_2, \cdots, j_m \neq \ell} r^{-m-1} a_{ij_1} a_{j_1 j_2} \cdots a_{j_m \ell}\right), \quad w(\ell) \neq 0, \tag{S.2}$$

where ℓ *is in* $1 \le \ell \le n$*.*

Proof. Here, we confirm five properties for irreducible non-negative matrices from the Perron–Frobenius theorem [1].

1. The spectral radius $\Lambda(\mathbf{A})$ of the matrix $\mathbf{A}$ coincides with a real eigenvalue $r = \Lambda(\mathbf{A})$.
2. The eigenvalue r is positive-real and simple.
3. If several eigenvalues of $\mathbf{A}$ have the same absolute value as r, they are complex eigenvalues
4. Both the left and right eigenspaces for $\mathbf{A}$ associated with r are one-dimensional.
5. It is only the left and right eigenvectors, $\mathbf{v}$ and $\mathbf{w}$, corresponding to r that all components of an eigenvector are strictly positive, in all eigenvectors for $\mathbf{A}$.

Because of the definition of the right eigenvector, the right eigenvector $\mathbf{w}$ corresponding to r satisfies

$$\mathbf{w} = \frac{1}{r}\mathbf{A}\mathbf{w}. \tag{S.3}$$

Because the eigenspace of $\mathbf{A}$ associated with r is one-dimensional, we can take the ℓ-th component of $\mathbf{w}$, $w(\ell)$, as an arbitrary constant. The right eigenvector $\mathbf{w}$ with a fixed $w(\ell)$ is uniquely determined, and the other components $w(i)$ $(i \neq \ell)$ obey

$$w(i) = \sum_{j=1}^{n} \frac{a_{ij}}{r} w(j) = \sum_{j \neq \ell} \frac{a_{ij}}{r} w(j) + \frac{a_{i\ell}}{r} w(\ell). \tag{S.4}$$

Furthermore, we redefine two $(n-1)$ column vectors $\mathbf{w}_\ell$, $\mathbf{a}_\ell$, and a matrix $\mathbf{A}_\ell$ as

$$\mathbf{w}_\ell := \begin{pmatrix} w(1) & \cdots & w(\ell-1) & w(\ell+1) & \cdots & w(n) \end{pmatrix}^\top, \tag{S.5}$$

$$\mathbf{a}_\ell := \begin{pmatrix} \frac{a_{1\ell}}{r} & \cdots & \frac{a_{\ell-1\ell}}{r} & \frac{a_{\ell+1\ell}}{r} & \cdots & \frac{a_{n\ell}}{r} \end{pmatrix}^\top, \tag{S.6}$$

$$\mathbf{A}_\ell := \left(\frac{a_{ij}}{r}\right)_{1 \le i,j \neq \ell \le n}. \tag{S.7}$$

The vector consisting of the components of the right eigenvector, except for $w(\ell)$, is the solution of

$$\mathbf{w}_\ell = \mathbf{A}_\ell \mathbf{w}_\ell + w(\ell)\mathbf{a}_\ell. \tag{S.8}$$

Because $\mathbf{a}_\ell$ is not a zero vector, the following inequality is valid.

$$\mathbf{w}_\ell > \mathbf{A}_\ell \mathbf{w}_\ell. \tag{S.9}$$

The inequality in Eq. (S.9) implies

$$\sum_{i\neq\ell} w(i) > \sum_{i\neq\ell}\sum_{j\neq\ell} \frac{a_{ij}}{r} w(j), \tag{S.10}$$

because all components of $\mathbf{w}_\ell$ are positive. Here, let a positive real number $r_\ell > 0$ be an eigenvalue of $\mathbf{A}_\ell$ that is equivalent to the spectral radius of $\mathbf{A}_\ell$. Then, a non-negative left eigenvector $\mathbf{v}_\ell^*$ exists corresponding to r_ℓ. Multiplying both sides of Eq. (S.9) by $\mathbf{v}_\ell^*$, it verifies that r_ℓ is always less than one.

$$\mathbf{v}_\ell^* \mathbf{w}_\ell > \mathbf{v}_\ell^* \mathbf{A}_\ell \mathbf{w}_\ell = r_\ell \mathbf{v}_\ell^* \mathbf{w}_\ell > 0 \implies 1 > r_\ell. \tag{S.11}$$

From Eq. (S.11), the solution of Eq. (S.8) with respect to $\mathbf{w}_\ell$ is given by

$$\mathbf{w}_\ell = w(\ell) \left(\mathbf{I}_\ell - \mathbf{A}_\ell\right)^{-1} \mathbf{a}_\ell, \tag{S.12}$$

where $\mathbf{I}_\ell$ denotes the $(n-1)\times(n-1)$ identity matrix. Similarly, because the spectral radius of $\mathbf{A}_\ell$ is less than one, the following representation of the inverse matrix $(\mathbf{I}_\ell - \mathbf{A}_\ell)^{-1}$ is justified.

$$\left(\mathbf{I}_\ell - \mathbf{A}_\ell\right)^{-1} = \sum_{m=0}^{\infty} \mathbf{A}_\ell^m. \tag{S.13}$$

The series on the right-hand side of Eq. (S.13) corresponds to the Neumann series. Substituting Eq. (S.13) into Eq. (S.12), the components of the right eigenvector $w(i)$, except for $w(\ell)$, become

$$w(i) = w(\ell)\left(r^{-1}a_{i\ell} + \sum_{m=1}^{\infty} \sum_{j_1,j_2,\cdots,j_m\neq\ell} r^{-m-1} a_{ij_1}a_{j_1j_2}\cdots a_{j_m\ell}\right), \quad i\neq\ell. \tag{S.14}$$

Furthermore, from Eq. (S.3), $w(\ell)$ should satisfy

$$w(\ell) = \sum_{j=1}^{n} r^{-1} a_{\ell j} w(j). \tag{S.15}$$

Upon substituting Eq. (S.14) into Eq. (S.15), we obtain

$$w(\ell) = w(\ell)\left(r^{-1}a_{\ell\ell} + \sum_{m=1}^{\infty} \sum_{j_1,j_2,\cdots,j_m\neq\ell} r^{-m-1} a_{\ell j_1}a_{j_1j_2}\cdots a_{j_m\ell}\right), \tag{S.16}$$

which yields

$$1 = r^{-1}a_{\ell\ell} + \sum_{m=1}^{\infty} \sum_{j_1,j_2,\cdots,j_m\neq\ell} r^{-m-1} a_{\ell j_1}a_{j_1j_2}\cdots a_{j_m\ell}. \tag{S.17}$$

The eigenvalue r must satisfy Eq. (S.17). It is another representation of the characteristic polynomial of $\mathbf{A}$, which is proved by the theorem in Text B. Therefore, all components of $\mathbf{w}$ have the following representation.

$$w(i) = w(\ell)\left(r^{-1}a_{i\ell} + \sum_{m=1}^{\infty} \sum_{j_1,j_2,\cdots,j_m\neq\ell} r^{-m-1} a_{ij_1}a_{j_1j_2}\cdots a_{j_m\ell}\right), \quad 1\leq i\leq n. \tag{S.18}$$

This completes the proof. □

Text B

Theorem for infinite series expansion of characteristic equation

Theorem B. *Let* $\mathbf{A} := (a_{ij})_{1\le i,j\le n}$ *be an irreducible non-negative matrix, and let* $\mathbf{I}$ *be an* $n \times n$ *identity matrix. The following series consists of entries and the dominant eigenvalue* r *in* $\mathbf{A}$*:*

$$-1 + r^{-1} a_{\ell\ell} + \sum_{m=1}^{\infty} \sum_{j_1, j_2, \cdots, j_m \neq \ell} r^{-m-1} a_{\ell j_1} a_{j_1 j_2} \cdots a_{j_m \ell} \tag{S.19}$$

is identical to the characteristic polynomial

$$\det\left(\mathbf{I} - \frac{1}{r}\mathbf{A}\right), \tag{S.20}$$

for all ℓ*.*

Proof. Recall that $\mathbf{w}_\ell$ is given by Eq. (S.12). The inverse matrix $(\mathbf{I}_\ell - \mathbf{A}_\ell)^{-1}$ is composed of $\det(\mathbf{I}_\ell - \mathbf{A}_\ell)$ and a cofactor matrix deleted ℓ-th row and ℓ-th column $\mathbf{D}_\ell := \left(\Delta_{ij}^{\ell\ell}\right)^{\top}_{1\le ij\neq\ell\le n}$ such that

$$(\mathbf{I}_\ell - \mathbf{A}_\ell)^{-1} = \frac{\mathbf{D}_\ell}{\det(\mathbf{I}_\ell - \mathbf{A}_\ell)}. \tag{S.21}$$

Each cofactor $\Delta_{ij}^{\ell\ell}$ is given by

$$\Delta_{ij}^{\ell\ell} = \begin{cases} (-1)^{i+j} d_{ij}^{\ell\ell} & i, j < \ell \\ (-1)^{i-1+j} d_{ij}^{\ell\ell} & j < \ell < i \\ (-1)^{i+j-1} d_{ij}^{\ell\ell} & i < \ell < j \\ (-1)^{i+j-2} d_{ij}^{\ell\ell} & \ell < i, j \end{cases}, \tag{S.22}$$

where each minor $d_{ij}^{\ell\ell}$ represents

$$d_{ij}^{\ell\ell} = \begin{vmatrix} 1 - \frac{a_{11}}{r} & \cdots & -\frac{a_{1j-1}}{r} & -\frac{a_{1j+1}}{r} & \cdots & -\frac{a_{1\ell-1}}{r} & -\frac{a_{1\ell+1}}{r} & \cdots & -\frac{a_{1n}}{r} \\ \vdots & \ddots & \vdots & \vdots & \cdots & \vdots & \vdots & \cdots & \vdots \\ -\frac{a_{\ell-11}}{r} & \cdots & \ddots & \vdots & \cdots & \vdots & \vdots & \cdots & \vdots \\ -\frac{a_{\ell+11}}{r} & \cdots & \vdots & \ddots & \cdots & \vdots & \vdots & \cdots & \vdots \\ \vdots & \cdots & \vdots & \vdots & \ddots & \vdots & \vdots & \cdots & \vdots \\ -\frac{a_{i-11}}{r} & \cdots & \vdots & \vdots & \cdots & \ddots & \vdots & \cdots & \vdots \\ -\frac{a_{i+11}}{r} & \cdots & \vdots & \vdots & \cdots & \vdots & \ddots & \cdots & \vdots \\ \vdots & \cdots & \vdots & \vdots & \cdots & \vdots & \vdots & \ddots & \vdots \\ -\frac{a_{n1}}{r} & \cdots & \vdots & \vdots & \cdots & \vdots & \vdots & \cdots & 1 - \frac{a_{nn}}{r} \end{vmatrix}. \tag{S.23}$$

Eq. (S.23) represents a case of $\ell < i, j$. Substituting Eq. (S.21) into Eq. (S.12), the component $w(j)$ $(j \neq \ell)$ of the right eigenvector w corresponding to r becomes

$$w(j) = w(\ell) \det(\mathbf{I}_\ell - \mathbf{A}_\ell)^{-1} \left(\sum_{i=1}^{\ell-1} \frac{a_{i\ell}}{r} \Delta_{ij}^{\ell\ell} + \sum_{i=\ell+1}^{n} \frac{a_{i\ell}}{r} \Delta_{ij}^{\ell\ell} \right). \tag{S.24}$$

Because of

$$\Delta_{ij}^{\ell\ell} = (-1)^{2\ell}\,\Delta_{ij}^{\ell\ell}, \tag{S.25}$$

Eq. (S.24) is equivalent to a cofactor expansion of the minor d_j^ℓ with respect to the ℓ-th column,

$$d_j^\ell = \sum_{i=1}^{\ell-1} \frac{-a_{i\ell}}{r}\,(-1)^{i+\ell}\,d_{ij}^{\ell\ell} + \sum_{i=\ell+1}^{n} \frac{-a_{i\ell}}{r}\,(-1)^{i+\ell-1}\,d_{ij}^{\ell\ell}, \tag{S.26}$$

where d_j^ℓ is a minor of the matrix $\mathbf{I} - \frac{1}{r}\mathbf{A}$ with respect to the ℓ-row and the j-th column such that

$$d_j^\ell := \begin{vmatrix} 1-\frac{a_{11}}{r} & \cdots & -\frac{a_{1j-1}}{r} & -\frac{a_{1j+1}}{r} & \cdots & -\frac{a_{1n}}{r} \\ \vdots & \ddots & \vdots & \vdots & \cdots & \vdots \\ -\frac{a_{\ell-11}}{r} & \cdots & \ddots & \vdots & \cdots & \vdots \\ -\frac{a_{\ell+11}}{r} & \cdots & \vdots & \ddots & \cdots & \vdots \\ \vdots & \cdots & \vdots & \vdots & \ddots & \vdots \\ -\frac{a_{n1}}{r} & \cdots & \vdots & \vdots & \cdots & 1-\frac{a_{nn}}{r} \end{vmatrix}. \tag{S.27}$$

Then, Eq. (S.24) is rewritten as

$$w(j) = \begin{cases} w(\ell)\det\left(\mathbf{I}_\ell - \mathbf{A}_\ell\right)^{-1}(-1)^{j+\ell}\,d_j^\ell & j < \ell \\ w(\ell)\det\left(\mathbf{I}_\ell - \mathbf{A}_\ell\right)^{-1}(-1)^{j+\ell-1}\,d_j^\ell & j > \ell \end{cases}. \tag{S.28}$$

Substituting Eq. (S.28) into the equation that $w(\ell)$ obeys,

$$w(\ell) = r^{-1}a_{\ell\ell}w(\ell) + \sum_{j\neq\ell} r^{-1}a_{\ell j}w(j), \tag{S.29}$$

let us consider the following deformation.

$$\begin{aligned} w(\ell) &= w(\ell)r^{-1}a_{\ell\ell} + w(\ell)\det\left(\mathbf{I}_\ell - \mathbf{A}_\ell\right)^{-1} r^{-1} \\ &\qquad \times \left(\sum_{j=1}^{\ell-1} a_{\ell j}\,(-1)^{j+\ell}\,d_j^\ell + \sum_{j=\ell+1}^{n} a_{\ell j}\lambda\,(-1)^{j+\ell-1}\,d_j^\ell\right) \\ \Longleftrightarrow 1 &= r^{-1}a_{\ell\ell} + \det\left(\mathbf{I}_\ell - \mathbf{A}_\ell\right)^{-1} r^{-1} \\ &\qquad \times \left(\sum_{j=1}^{\ell-1} a_{\ell j}\,(-1)^{j+\ell}\,d_j^\ell + \sum_{j=\ell+1}^{n} a_{\ell j}\,(-1)^{j+\ell-1}\,d_j^\ell\right) \\ \Longleftrightarrow 0 &= -1 + r^{-1}a_{\ell\ell} + \det\left(\mathbf{I}_\ell - \mathbf{A}_\ell\right)^{-1} r^{-1} \\ &\qquad \times \left(\sum_{j=1}^{\ell-1} a_{\ell j}\,(-1)^{j+\ell}\,d_j^\ell + \sum_{j=\ell+1}^{n} a_{\ell j}\,(-1)^{j+\ell-1}\,d_j^\ell\right) \\ \Longleftrightarrow 0 &= \left(1 - r^{-1}a_{\ell\ell}\right)\det\left(\mathbf{I}_\ell - \mathbf{A}_\ell\right) \\ &\qquad + \sum_{j=1}^{\ell-1} -r^{-1}a_{\ell j}\,(-1)^{j+\ell}\,d_j^\ell + \sum_{j=\ell+1}^{n} -r^{-1}a_{\ell j}\,(-1)^{j+\ell-1}\,d_j^\ell. \end{aligned} \tag{S.30}$$

Because $(-1)^{j+\ell} d_j^\ell$ is equivalent to a cofactor of $\det\left(\mathbf{I}-\frac{1}{r}\mathbf{A}\right)$ for the ℓ-th row and j-th column, Eq. (S.30) is nothing but a cofactor expansion of $\det\left(\mathbf{I}-\frac{1}{r}\mathbf{A}\right)$ with respect to the ℓ-th row.

$$\det\left(\mathbf{I}-\frac{1}{r}\mathbf{A}\right) = \sum_{j=1}^{\ell-1} -r^{-1}a_{\ell j}\,(-1)^{j+\ell}\,d_j^\ell + \left(1-r^{-1}a_{\ell\ell}\right)(-1)^{2\ell}\,d_\ell^\ell + \sum_{j=\ell+1}^{n} -r^{-1}a_{\ell j}\,(-1)^{j+\ell-1}\,d_j^\ell = 0. \quad \text{(S.31)}$$

Therefore, the claim of the theorem is true. □

Text C

Original definition of type-reproduction number

Let us introduce a region-specific index called the type reproduction number, different from the R_0. Supposing a division of prefectures into two appropriate areas 1 and 2 (for example, urban and rural areas), the rearranged matrix $\Phi(\lambda)$ of $\Psi(\lambda)$ contains the following four leading submatrices.

$$\mathbf{\Phi}(\lambda) = \begin{pmatrix} \mathbf{Q}_{11} & \mathbf{Q}_{12} \\ \mathbf{Q}_{21} & \mathbf{Q}_{22} \end{pmatrix}. \quad \text{(S.32)}$$

$\mathbf{Q}_{k\ell}$ denotes the transition matrix from area ℓ to area k. When area 1 holds κ prefectures, $\mathbf{Q}_{11}$ becomes a $\kappa\times\kappa$ matrix, and $\mathbf{Q}_{22}$ becomes $(n-\kappa)\times(n-\kappa)$ matrix. Similarly, $\mathbf{Q}_{12}$ is an $\kappa\times(n-\kappa)$ matrix, and $\mathbf{Q}_{21}$ is the $(n-\kappa)\times\kappa$ matrix. Suppose that area 1 is the target area under the spectral radius of $\mathbf{Q}_{22}$, which is less than one.

$$\Lambda(\mathbf{Q}_{22}) < 1. \quad \text{(S.33)}$$

Let an $n\times n$ projection matrix $\mathbf{U}$ represent the internal migration within the target area.

$$\mathbf{U} := \begin{pmatrix} \mathbf{I}_{11} & \mathbf{O}_{12} \\ \mathbf{O}_{21} & \mathbf{O}_{22} \end{pmatrix}, \quad \text{(S.34)}$$

where $\mathbf{I}_{11}$ is an identity matrix and $\mathbf{O}_{k\ell}$ is a zero matrix, which coincide with each index of $\mathbf{Q}_{k\ell}$ in $\mathbf{\Phi}(\lambda)$. Substituting $\lambda = 1$ into $\mathbf{\Phi}(\lambda)$, $\Lambda(\mathbf{Q})$ gives the definition of $\mathcal{T}_j$ in area 1 [2], where

$$\mathbf{Q} := \mathbf{U}\mathbf{\Phi}(\lambda)\left(\mathbf{I}-(\mathbf{I}-\mathbf{U})\,\mathbf{\Phi}(\lambda)\right)^{-1}\Big|_{\lambda=1}. \quad \text{(S.35)}$$

By computing Eq. (S.35) on the right-hand side, we obtain

$$\mathbf{Q} = \begin{pmatrix} \mathbf{Q}_1 & \mathbf{Q}_{12}\left(\mathbf{I}-\mathbf{Q}_{22}\right)^{-1} \\ \mathbf{O}_{21} & \mathbf{O}_{22} \end{pmatrix}, \quad \text{(S.36)}$$

$$\mathbf{Q}_1 := \mathbf{Q}_{11} + \mathbf{Q}_{12}\left(\mathbf{I}-\mathbf{Q}_{22}\right)^{-1}\mathbf{Q}_{21}, \quad \text{(S.37)}$$

where $\mathbf{I}$ is an $(n-\kappa)\times(n-\kappa)$ identity matrix. Then, we obtain $\Lambda(\mathbf{Q}) = \Lambda(\mathbf{Q}_1)$. For $\Lambda(\mathbf{Q}_1)$, the relationship between other indicators is valid [3]:

$$\operatorname{sgn}\left(\Lambda(\mathbf{Q}_1)-1\right) = \operatorname{sgn}\left(\rho-1\right) = \operatorname{sgn}\left(\lambda_1-1\right). \quad \text{(S.38)}$$

This definition serves as an indicator if and only if the spectral radius of $\mathbf{Q}_{22}$ is less than one ($\Lambda(\mathbf{Q}_{22}) < 1$).

Text D

Extention theorem of type-reproduction number

Theorem D. *Let $g_j(y)$ be given by Eq. (35), and $\lambda_1 \in \mathbb{R}_+ \setminus \{0\}$ be the dominant eigenvalue satisfying Eq. (18). Then, $g_j(1) \leq 1$ is equivalent to $\lambda_1 \leq 1$, and $1 < g_j(1) \leq \infty$ in $\lambda_1 > 1$ for all $1 \leq j \leq n$.*

Proof. Let the M-th partial sums of $g_j(y)$ be $g_j^M(y)$, defined as

$$g_j^M(y) := \psi_{jj}(y) + \sum_{m=1}^{M} \psi_{jj}^{(m)}(y). \tag{S.39}$$

Because of the inequality $\frac{d}{dy}\psi_{ij}(y) < 0$ (cf. Eq. (17)), $g_j^M(y)$ is positive and monotonically decreases in y for all M. Therefore,

$$\frac{d}{dy} g_j^M(y) < 0. \tag{S.40}$$

Noting that $g_j(y)$ is convergent in $y \geq \lambda_1$, the following inequality holds.

$$\lim_{M\uparrow\infty} \frac{d}{dy} g_j^M(y) = \frac{d}{dy} g_j(y) < 0, \quad j = 1, 2, \ldots, n. \tag{S.41}$$

Because $g_j(\lambda_1) = 1$ for all j, we have $g_j(y) \leq 1$ for $\lambda_1 \leq y$. However, if there exists an $M_0 \geq 0$ such that

$$g_j^M(1) > 1 = g_j(\lambda_1), \text{ for all } M \geq M_0, \tag{S.42}$$

then $\lambda_1 > 1$ because of the monotonicity of $g_j^M(y)$ in y and $g_j^M(y) \leq g_j(y)$ (for example, if $\psi_{jj}(1) > 1$, then $M_0 = 0$). Therefore, $g_j(1) \leq 1 = g_j(\lambda_1)$ in $\lambda_1 \leq 1$, and $1 < g_j(1)$ in $1 < \lambda_1$ for all j. □

References

1. Meyer CD. Matrix analysis and applied linear algebra. vol. 71. Siam; 2000.
2. Heesterbeek J, Roberts M. The type-reproduction number T in models for infectious disease control. Mathematical biosciences. 2007;206(1):3–10.
3. Inaba H. The net reproduction rate and the type-reproduction number in multiregional demography. Vienna Yearbook of Population Research. 2009; p. 197–215.
